# Do increases in the price of fuel increase levels of fuel theft? Evidence from England and Wales

**DOI:** 10.1186/s40163-023-00182-7

**Published:** 2023-03-22

**Authors:** Aiden Sidebottom, Iain Agar, Justin Kurland

**Affiliations:** 1 Jill Dando Institute of Security and Crime Science, University College London, London, UK; 2 London Metropolitan Police Service, London, UK; 3 Independent Scholar, Brattleboro, USA

**Keywords:** Bilking, Drive-offs, Fuel, Petrol, Price, Theft

## Abstract

Fuel prices have increased sharply over the past year. In this study we test the hypothesis that increases in the price of fuel are associated with increases in motorists filling their fuel tank and driving off without paying. We use weekly crime data from six police forces in England and Wales for the period January 2018 to July 2022, combined with regional data on the number of fuel sales and average fuel prices. Our results demonstrate an overall weak price-theft relationship for the 238 week study period, less so than in previous studies. However, we find strong evidence that the recent spike in fuel prices was associated with elevated levels of fuel theft. The implications of our findings for future research and crime prevention are discussed.

## Introduction

The price of fuel has increased sharply over the past year. In the UK, for example, fuel prices rose by 43.7% in the year to July 2022 (Office of National Statistics, 2022), with some fuel retailers surpassing the ‘previously unthinkable average of £2 a litre’ (Burrell, 2022). These price increases are attributed to three main drivers: surges in demand for fuel following the COVID-19 pandemic, supply shortages associated with the Russian invasion of Ukraine, and weaknesses in the pound against the dollar (Tooze, 2022).^1^

In response to soaring fuel costs, alongside rising food and energy prices (Francis-Devine et al., 2022), many UK news agencies have warned of increases in motorists filling their fuel tanks and driving off without paying (for e.g. BBC, 2022a, 2022b; Guardian, 2022), a type of theft variously known as ‘bilking’, ‘gasoline drive-offs’ or ‘gas-and-dash’ (see LaVigne, 1994; Meini & Clarke, 2012). Prior studies lend support to these claims. Moffatt and Fitzgerald (2006) found a strong correlation between the price of petrol and levels of petrol theft in New South Wales (Australia).^2^ Likewise in England, Draca et al. (2019) found a statistically significant fuel price-theft relationship. Several studies report similar findings for other commodities and consumer goods, particularly metals (Brabenec & Montag, 2018; Kirchmaier et al., 2020; Mares & Blackburn, 2017; Posick et al., 2012; Quinn et al., 2022; Sidebottom et al., 2011, 2014). These findings are typically interpreted using an economic (Becker, 1968) or rational choice framework (Cornish & Clarke, 1987, 2017) as evidence that offenders are responsive to changes in market prices, and that all things being equal, more financially rewarding theft targets are more attractive theft targets.

Informed by previous research, the current study tests the hypothesis that recent increases in the price of fuel are associated with increases in bilking from UK petrol stations.

### Data

Data come from three sources. The first dataset comprised weekly counts of bilking incidents recorded by six police forces in England and Wales between January 2018 and July 2022. In total, there were 36,573 police recorded bilking incidents over the 238 week study period (mean count per week = 153.7, SD = 46.0).^3^ The second dataset comprised regional average weekly fuel sales compiled and published by the UK Government Department for Business, Energy & Industrial Strategy.^4^ These data were used here as the denominator to compute a bilking rate per 100,000 fuel sales. We opted to use a bilking *rate* as our dependent variable (as opposed to the count of bilking offences) to account for fluctuations in traffic volume over time, most notably the significant falls in vehicle traffic associated with COVID-19 lockdown restrictions (Department for Transport, 2021). The third dataset used here was also published by the Department for Business, Energy & Industrial Strategy, and comprised weekly national average fuel prices.^5^For this study, we used a combined average price estimate of diesel and unleaded petrol.

### Analysis

Analysis comprises six steps. First, we present the (unadjusted) trends in fuel price and bilking rate over time. To better enable comparisons across different datasets, we then normalize both time series by computing the percentage of weekly change in average fuel price and bilking rates. To investigate the fuel price-theft relationship, we then calculate the Spearman correlation coefficient to determine whether weekly percentage changes in price and theft co-vary over the 238 week study period. This approach provides a single global measure of the petrol price-theft relationship; it does not account for shorter temporal periods where the relationship between fuel price and bilking may vary. To assess this, we next calculate Spearman correlation coefficients for successive rolling windows of shorter time periods, in our case sixty 4 week (1 month) intervals.

The fifth step explores directionality between the two-time series. To do this we use a time lagged cross-correlation approach, which enables us to determine whether changes in the price of petrol lead to changes in rates of petrol theft and, if so, at what point over the study period (238 weeks) do we observe maximum correlation.

Finally, we use a statistical technique that is growing in popularity in the field of signal processing, known as ‘instantaneous phase synchrony’ (see Pedersen et al., 2018). We use this technique to determine the specific time points (here weeks) where price and theft exhibit the greatest correlation. Phase, in the context of this analysis, refers to the angle of the time series (signals) calculated through a Hilbert transform (Mormann et al., 2000). When there is alignment between, here, price and theft, the angular difference becomes zero and coherence is then quantified by subtracting this difference from one. The advantage of using instantaneous phase synchrony analysis is that, unlike the previous correlation-based sliding window analysis, there is no requirement to pre-specify the time-period over which correlation coefficients are calculated. Rather, this approach enables single time-point resolution for the comparison of signals, again something that is not possible with correlation-based analysis where more than one point is required to estimate a coefficient. All analysis source code (in Python) are available at https://osf.io/235sx/ for replication and scrutiny.^6^

## Results

**Fig. 1:**
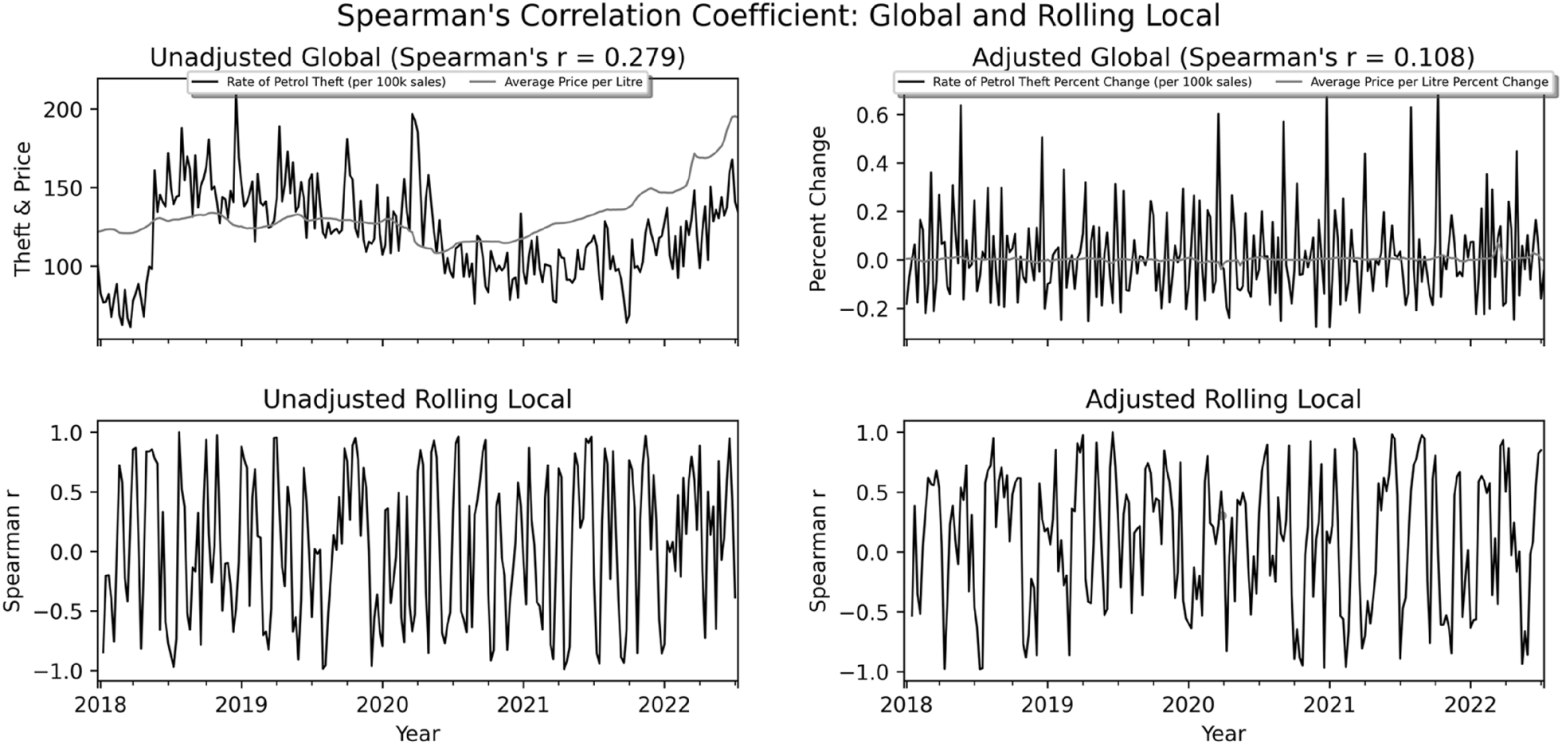
Unadjusted and adjusted global and rolling local correlations of mean fuel price and bilking rate per 100,000 fuel sales, Dec 2017 to July 2022

The left upper panel of Fig. 1 displays the (unadjusted) trends in the average price per litre of fuel (pence) and the rate of police recorded bilking offences per 100,000 fuel sales (*r* = 0.279, *p* < 0.001). The right upper panel shows the (adjusted) week-on-week percentage change in price and bilking over the same time period (*r* = 0.108, *p* = 0.098). Both panels show that fuel prices in 2018 and 2019 remained fairly stable at around 120–130 pence per litre. A fall in the cost of fuel occurred in early 2020, coinciding with the implementation of COVID-19 lockdown restrictions. After this point, fuel costs rose significantly, reaching nearly £2 per litre in mid 2022. In terms of crime, rates of bilking, like many crime types (see Langton et al., 2021), declined considerably in early 2020 and remained comparatively low for much of 2021. From late 2021 onwards, the bilking rate exhibits a general upward trend, reaching pre-COVID levels by mid-2022.^7^

Visual inspection of the upper left panel (unadjusted) of Fig. 1 suggests that prior to the COVID-19 pandemic, UK fuel prices were generally stable and showed limited convergence with the more volatile bilking time series. By contrast, the rising fuel costs observed from 2021 onwards show a similar trajectory to that of bilking offences. Further analysis is thus warranted.

As described above, to investigate the fuel price-theft relationship, we normalised the two time-series by computing the percentage weekly change in price and theft. We then calculated the global (adjusted) Spearman’s correlation coefficient for the entire 238 week study period. The Spearman’s correlation coefficient of 0.108 (p-value = 0.098) indicates a weak and statistically non-significant correlation between fuel price and bilking offences over time. This finding is inconsistent with previous studies which report a positive and statistically significant fuel price-theft relationship (see Draca et al., 2019; Moffatt & Fitzgerald, 2006).^8^

We then performed correlation-based sliding window analysis to investigate (again using Spearman’s correlation coefficients) the fuel price-theft relationship for sixty 4 week (1 month) intervals. The results of this analysis using both the unadjusted and adjusted time series are presented in the bottom left and right panels of Fig. 1. The pattern for both of these analyses bring to light a potentially more complex relationship with *r*-statistics oscillating between 1 and − 1. Such volatility is indicative of a kind of temporal ordering. That is, one time-series may follow the other thus obscuring the true nature of the relationship between, here, petrol prices and rates of petrol theft.

**Fig. 2:**
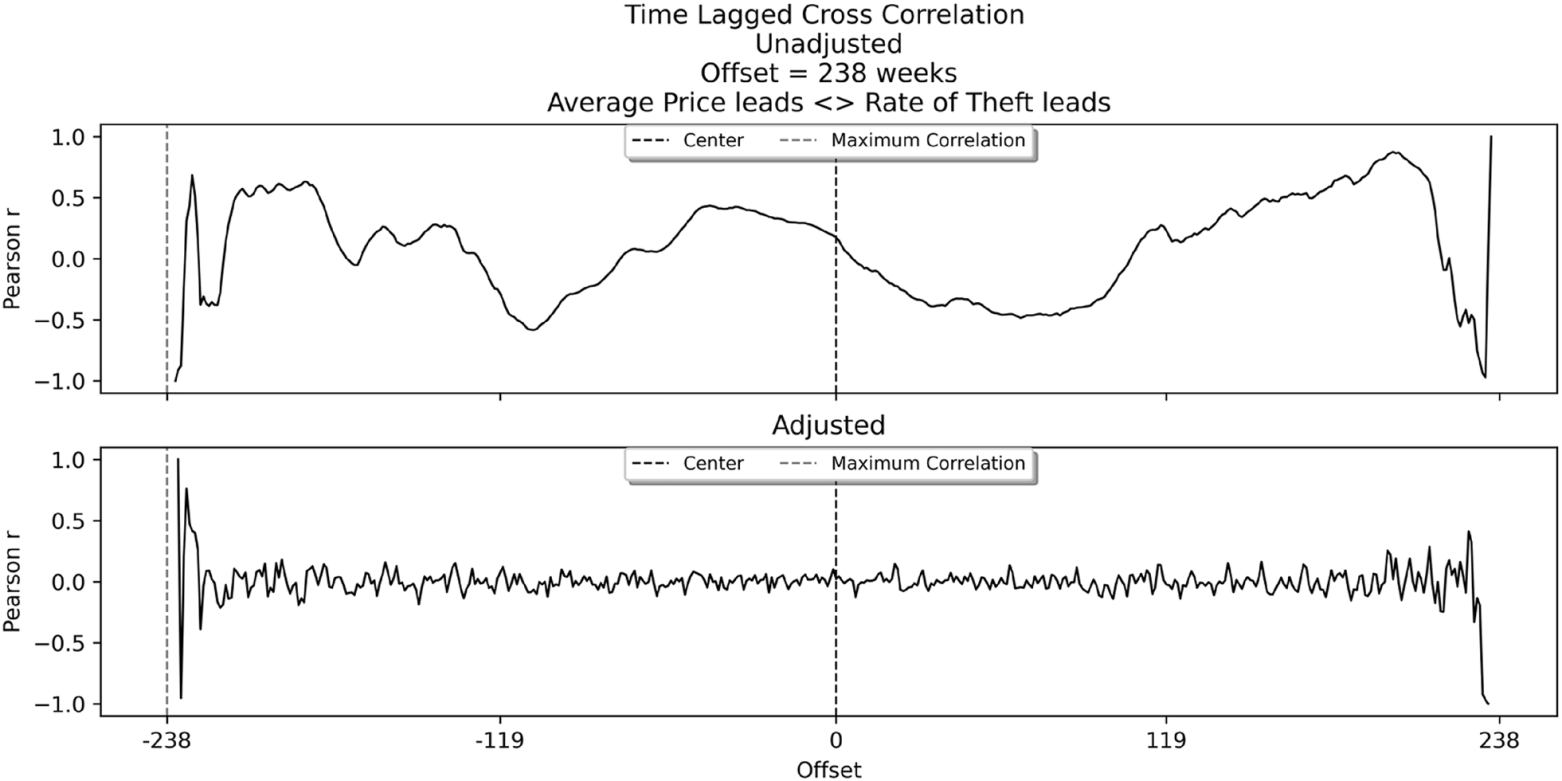
Unadjusted and adjusted time-lagged cross correlations of mean fuel price and bilking rate per 100,000 fuel sales, Dec 2017 to July 2022

To capture which time-series leads and which follows, a time-lagged cross-correlational analysis was undertaken. The results are shown in Fig. 2, again using both unadjusted (top panel) and adjusted (bottom panel) data. The left-side of both panels captures when price leads and the right-side captures when theft leads, while the dotted vertical grey line captures the precise moment that the correlation between the two time-series is greatest. Figure 2 reveals two important findings. First, in both the unadjusted and adjusted time-lagged cross correlation plots, price is found to lead theft, as evidenced by the negative offset (x-axis). Second, the time-window of maximum correlation between price and theft occurs at the very end of the study period: week 238.

**Fig. 3:**
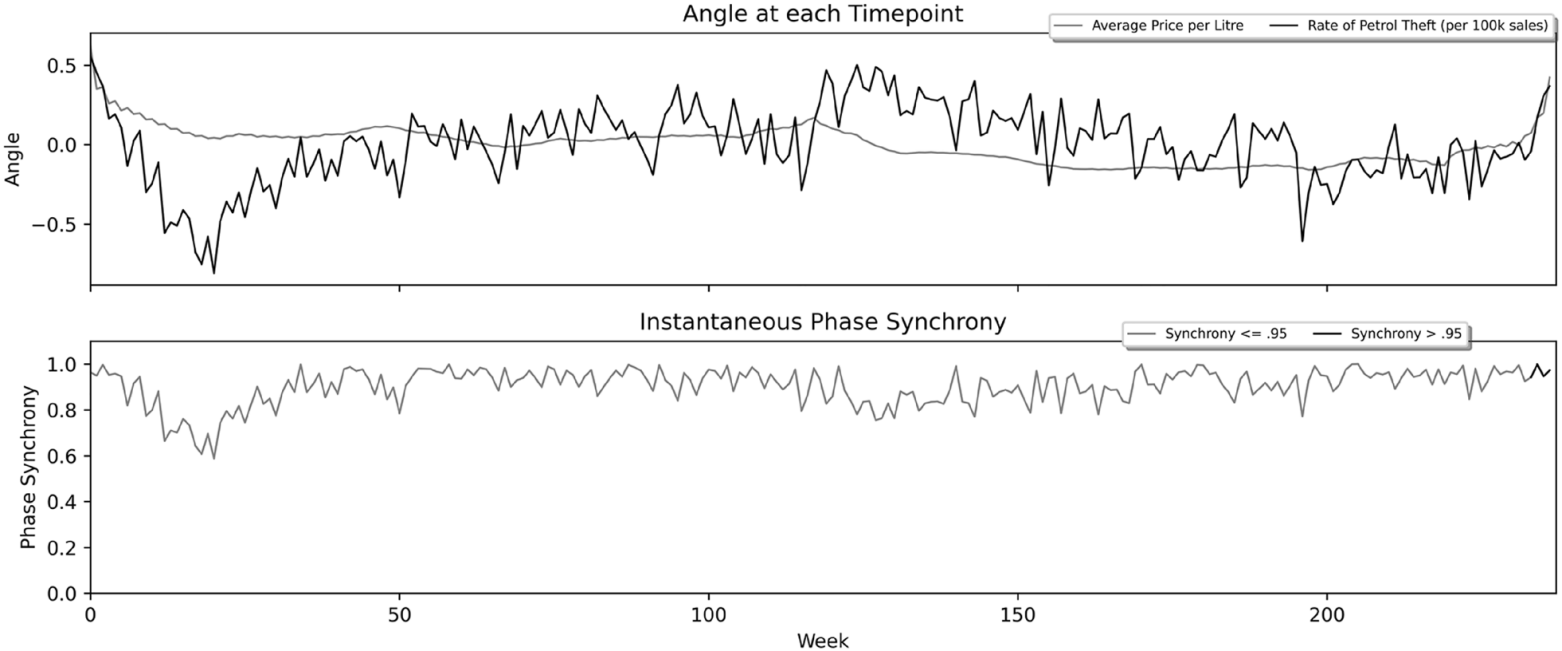
Instantaneous Phase Synchrony of mean fuel price and bilking rate per 100,000 fuel sales, Dec 2017 to July 2022

Finally, we used instantaneous phase synchrony analysis to compute moment-to-moment synchrony between the two time-series. As indicated above, phase synchrony ranges between zero and one, with lower values close to zero indicating little synchrony and values closer to one indicating higher synchrony. In Fig. 3, the top panel provides the precise phase angle (radians) of each respective timepoint across the time-series used to calculate phase synchrony in the bottom panel. Results indicate that the period across which the highest level of continuous synchrony occurred was the five-week period at the end of the time series—weeks 234 through 238—highlighted in black in the bottom panel and producing a near perfect synchrony score of above 0.95.

## Discussion

The past year has seen steep rises in the price of fuel. In the current study we tested the hypothesis that recent increases in the price of fuel have led to increases in fuel theft, measured here as motorists filling their fuel tanks and driving off without paying. Contrary to previous research (Draca et al., 2019; Moffatt & Fitzgerald, 2006), we found little evidence of a strong long-term price-theft relationship. During periods of relatively stable petrol prices, we found that changes in the cost of fuel had little impact on changes in the rate of bilking. However, when analysing weekly co-variance in price and theft, we found that the recent record highs in petrol prices *were* significantly associated with higher levels of bilking offences. This finding lends support for the claim that bilking offences have increased in response to soaring petrol prices.

It is unclear why the results of this study differ to those of previous research. Differences in study site, time period and analytical approaches are all possible explanations. Moreover, different to Moffatt and Fitzgerald (2006) and Draca et al. (2019), the data used in our study cover the COVID-19 period which, as we have discussed and sought to account for, caused major disruption in traffic volume, fuel sales and criminal activity, amongst other things. Further research is hence needed to determine the generalisability of our findings.

This study contributes to the research literature on what factors influence the theft of petrol. Our findings suggest that exceedingly high petrol prices may act as an incentive for theft to those unwilling or unable to pay the increased prices. More generally, our findings provide further evidence that changes in the price of commodities affect levels of commodity theft (Draca et al., 2019; Quinn et al., 2022). We suggest that future studies, building on that reported here, might usefully explore three research questions. The first relates to tipping points. An original feature of this study was the use of a statistical technique from the field of signal processing: instantaneous phase synchrony analysis. An advantage of using this approach was that it enabled us to investigate the relationship between fuel price and theft over much shorter temporal intervals than in previous studies. Doing so suggested the presence of a tipping point: a price point at which two largely uncorrelated variables exhibited high levels of synchrony. Further research might helpfully explore the existence of tipping points for other desirable commodities and consumer goods, and look for similarities between them, such as the magnitude and/or speed of price increases. Discovery of such tipping points would clearly be of practical use for the police and partners as indicators of an expected surge in theft offences.

A second research question concerns the distribution of bilking incidents across petrol stations. It is well-established that crime is unevenly distributed across comparable facilities; most experience little or no crime and some experience a lot (Eck, Clarke & Guerette, 2007). Similar patterns have been observed for bilking in particular (see Chainey, 2021). Preliminary analysis of a dataset made available to the authors suggests a similar pattern is observed for the area and time-period covered in this study (see Appendix 1). More specifically, we find that 11% of petrol stations (n = 153) accounted for 50% of all police recorded bilking incidents (n = 17,907). Future research is needed to investigate what factors make some petrol stations more susceptible to bilking than others (see La Vigne, 1994). Moreover, research could usefully explore whether the recent increases in bilking concentrate in a small number of repeatedly targeted petrol stations, or whether the increase in petrol prices has led to more petrol stations being the victims of theft.

A third research question relates to who is stealing petrol, and why. Fuel differs to some other commonly stolen commodities (such as metals) in that fuel is generally stolen for consumption rather than resale. The recent rise in petrol prices has occurred at a time when many UK households and businesses are struggling to cope with rising living costs. It is therefore possible that the observed rise in bilking reflects crimes of need, committed by individuals with little if any prior involvement in crime and unable to pay the elevated petrol prices. An alternative hypothesis is that the rise in bilking offences is mainly attributed to experienced offenders taking advantage of available opportunities, who either baulk at paying higher petrol prices or who stand to profit from selling stolen fuel to others. An examination of the offending history of those charged with bilking might be revealing.^9^

Finally, it warrants mention that the crime of bilking is an offence which can and has been prevented. The installation of petrol pump pre-payment methods, for example, largely removes opportunities for driving off without payment. This is important to acknowledge given the potential harms associated with bilking, particularly the dangers to customers and staff when offenders flee from petrol forecourts at high speed (Meini & Clarke, 2012).^10^ In the UK, however, pre-payment methods are common but not mandatory. This likely reflects an effort on the part of fuel retailers to preserve the revenue generated from motorists visiting forecourt stores and purchasing items other than fuel. According to a 2018 report by the Association of Convenience Stores, UK forecourts generated £4.1 billion in sales excluded the sale of fuel. Assuming that petrol pump pre-payment methods remain discretionary, alternative ways to prevent bilking may include the use of CCTV cameras, automatic number plate recognition technology, redesigning petrol forecourts to maximise surveillance opportunities and/or elongating the time/distance between pumps and exit. Whilst the current study does not speak to the merits or appropriateness of different prevention methods, our findings, if generalisable, do suggest that the need for effective prevention methods is heightened during times of elevated prices, and that prevention gains could be maximised by targeting prevention methods at those petrol stations where drive-offs are more frequent.

## Figures and Tables

**Fig. 4:**
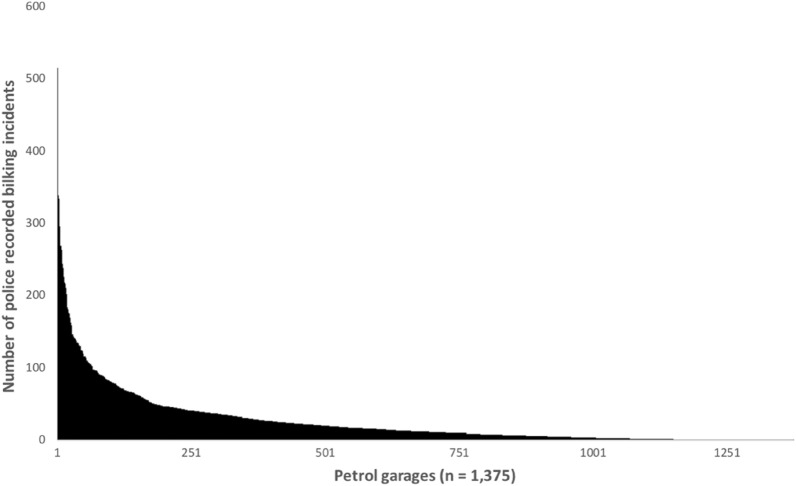
Distribution of police recorded bilking incidents (n = 35,729) across petrol garages (n = 1375) in six UK police force areas (Jan 2018 to July 2022)

## Data Availability

All analysis source code (in Python) are available at https://osf.io/235sx/.

## Do some petrol stations experience more petrol drive-offs than others?

Across the 6 police force areas covered in this study, there were 35,729 bilking incidents in which the location was recorded (98% of all offences). These incidents occurred at a total of 1375 petrol stations. Appendix 1 shows the distribution of bilking offences across these petrol stations. Consistent with previous research (Eck, Clarke and Guerette, 2007; Chainey, 2021), bilking incidents are found to be unevenly distributed across petrol stations. Most stations experience few drive-offs and a few are disproportionately affected. The most targeted petrol station reported 515 bilking offences. Across the entire study period, 11 percent of petrol stations (n = 153) accounted for half of all police recorded bilking incidents (n = 17,907).

See Fig. 4.

## Footnotes

1 To elaborate: the price of fuel has increased because the price of crude oil, which is used to make petrol and diesel, has increased. Increases in the price of crude oil are attributed to three main factors. The first is COVID-19. The lockdown restrictions implemented to curb the spread of COVID-19 resulted in large declines in vehicle usage and, by extension, demand for fuel (Department for Transport, 2021). As lockdown restrictions abated, vehicle usage rates increased and demand for fuel grew, but many fuel suppliers struggled to meet rising demand, with supply shortages leading to increases in price. Then came the Russia-Ukraine 2022 conflict. Numerous financial sanctions were imposed on Russia following their invasion of Ukraine. This included a refusal of many countries to purchase Russian oil. As the second-largest exporter of crude oil, reduced supply of Russian oil contributed to global supply shortages and in turn increases in oil price. Third, the crude oil used to make petrol and diesel is purchased in US dollars, and in recent months the British pound has been weak against the US dollar, thereby making fuel more expensive.

2 It should be noted that Moffatt and Fitzgerald used the broader dependent variable of ‘service station fraud’ of which the vast majority of incidents (83%) related to fuel theft.

3 Readers should note that the nature of the data provided – aggregated weekly counts of bilking – meant that we were unable to carry out analysis individually for the six police forces represented in our data.

4 https://www.gov.uk/government/statistics/average-road-fuel-sales-and-stock-levels.

5 https://www.gov.uk/government/statistics/weekly-road-fuel-prices

6 We note that a reviewer of this paper suggested a different analytical approach, one which more closely resembles the methods used in Draca et al. (2019). Instead of normalising the data by computing the percentage weekly change in average fuel price and bilking rates, this alternative approach involved log-transforming these variables and performing a regression of bilking on fuel price both for the log–log variables and the first differences of these logged variables. As a sensitivity check, we ran these suggested analyses. Reassuringly, the results were consistent with our preferred approach: log–log regression analysis generated a Pearson's r of 0.216 (p-value < 0.001) and the difference in log–log regression analysis generated a Pearson's r of 0.099 (p-value = 0.12). We thank the reviewer for suggesting this alternative approach.

7 Figure 1 also suggests a sudden jump in bilking offences in 2018. Despite enquiries made by the authors, it remains unclear whether this increase is a result of changes in reporting, recording or a real increase in crime, or a combination thereof. We note that this period coincides with when several participating police forces were transitioning to a new data management system. It is therefore possible that the lower number of bilking offences in early 2018 reflects the fact that not all forces were present on the system at this time. To investigate this, future research could usefully replicate our methods using data from police forces that did change their data management system.

8 To expand: Moffatt and Fitzgerald (2006) estimated a bivariate correlation coefficient of 0.90. Draca et al. (2019) reported a 12 month log-change price-theft correlation of 0.53. They also report various ordinary least squares and instrumental variables estimates between 0.6 and 0.7.

9 However, it should be noted that the solved rates for this type of offence in the UK are typically in the order of 1%. Any meaningful analyses of offenders would therefore require a large dataset covering a long time-period.

10 Readers may be interested to know that in British Columbia (Canada), a regulation was enacted in 2006 that requires urban petrol stations to implement mandatory pre-pay systems at night. This regulation is known as “Grant’s law”, and was proposed following the death in March 2005 of petrol station attendant Grant De Patie, who was struck and killed when trying to prevent a motorist from making off without paying for $12 of gasoline.
